# Tryptophan 2,3-dioxygenase is a key modulator of physiological neurogenesis and anxiety-related behavior in mice

**DOI:** 10.1186/1756-6606-2-8

**Published:** 2009-03-27

**Authors:** Masaaki Kanai, Hiroshi Funakoshi, Hisaaki Takahashi, Tomoko Hayakawa, Shinya Mizuno, Kunio Matsumoto, Toshikazu Nakamura

**Affiliations:** 1 Division of Molecular Regenerative Medicine, Department of Biochemistry and Molecular Biology, Osaka University Graduate School of Medicine, Osaka 565-0871, Japan; 2 Department of Molecular and Cellular Biology, School of Medicine, Ehime University, Ehime, Japan; 3 Department of Vascular Regeneration, Graduate School of Medicine, The University of Tokyo, Tokyo, Japan; 4 Division of Tumor Dynamics and Regulation, Kanazawa University Cancer Research Institute, Kanazawa, Japan; 5 Kringle Pharma Joint Research Division for Regenerative Drug Discovery, Osaka University, Osaka 565-0871, Japan

## Abstract

Although nutrients, including amino acids and their metabolites such as serotonin (5-HT), are strong modulators of anxiety-related behavior, the metabolic pathway(s) responsible for this physiological modulation is not fully understood. Regarding tryptophan (Trp), the initial rate-limiting enzymes for the kynurenine pathway of tryptophan metabolism are tryptophan 2,3-dioxygenase (TDO) and indoleamine 2,3-dioxygenase (IDO). Here, we generated mice deficient for *tdo *(*Tdo*^-/-^). Compared with wild-type littermates, *Tdo*^-/- ^mice showed increased plasma levels of Trp and its metabolites 5-hydroxyindoleacetic acid (5-HIAA) and kynurenine, as well as increased levels of Trp, 5-HT and 5-HIAA in the hippocampus and midbrain. These mice also showed anxiolytic modulation in the elevated plus maze and open field tests, and increased adult neurogenesis, as evidenced by double staining of BrdU and neural progenitor/neuronal markers. These findings demonstrate a direct molecular link between Trp metabolism and neurogenesis and anxiety-related behavior under physiological conditions.

## Background

**Figure 1:**
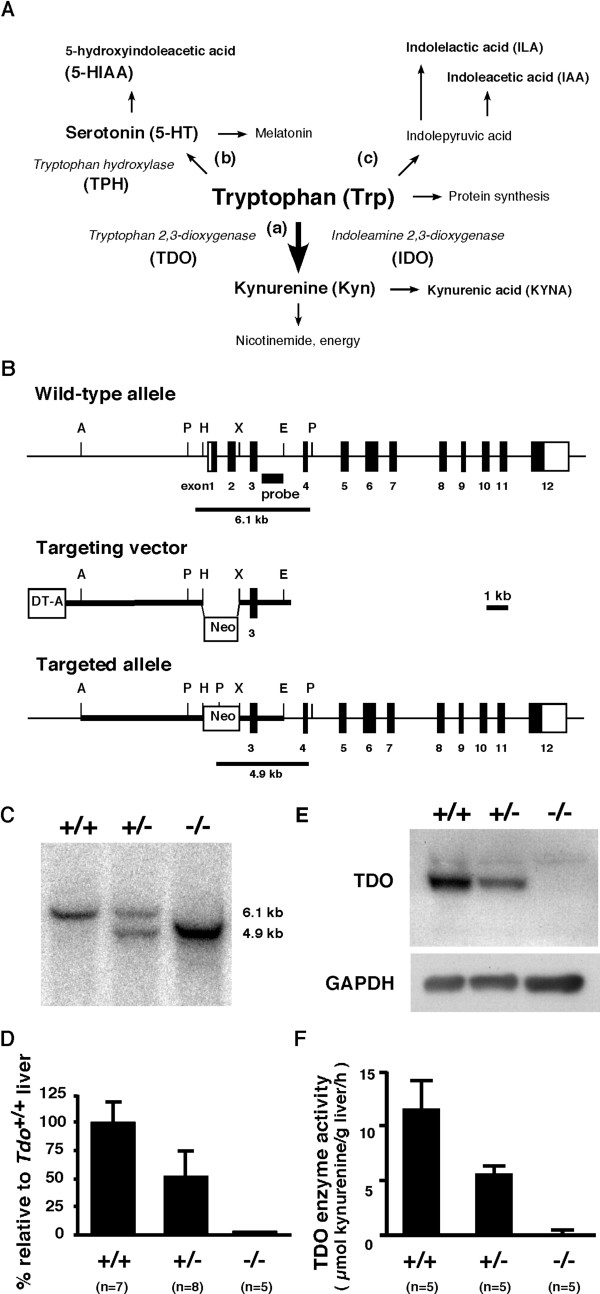
**Generation of *tdo*-deficient (*Tdo*^-/-^) mice**. (A) Schema of the Trp metabolic pathways. (a) the Kyn pathway. Over 95% of the dietary Trp is metabolized along this pathway. (b) the serotonin pathway. (c) the transamination pathway. (B) A targeting strategy for *tdo *gene disruption. Exons are represented as *numbered boxes *(coding regions; *black boxes*). The probe for Southern blot analysis is indicated by a *solid bar*. *Apa*I, A; *Pvu*II, P; *Hind*III, H; *Eco*RI, E; *Xba*I, X; *Neo*, PGK-neomycin resistant cassette; *DT-A*, diphtheria toxin-A. (C) Southern blot analysis of representative progeny. Tail genomic DNA was digested with *Pvu*II for hybridization with a specific probe against the intron sequence between exons 3 and 4 of *tdo*. The expected sizes of the hybridized DNA fragment for *Tdo*^+/+ ^(+/+) and *Tdo*^-/- ^(-/-) mice are 6.1 kb and 4.9 kb, respectively. (D) Quantitative real-time RT-PCR for *tdo *mRNA expression in adult liver. Mouse *tdo/gapdh *of adult liver in *Tdo*^+/+ ^mice was arbitrarily given a value of 100%. Values are means ± S.D. (E) Western blot analysis. Total liver homogenates were immunoblotted with TDO-specific antiserum. (F) Assay for TDO enzyme activity, determined in total liver lysates from 10-week-old animals of each genotype. Values represent means ± S.D.

Mental disorders and affective status, in particular depression and anxiety disorders, are increasingly important medical and social issues in the 21^st ^century [1]. Although associations with these conditions have been proposed for a wide range of factors, including obesity, which may play a role in them [2,3], the molecular mechanisms remain to be precisely elucidated. Modulatory roles have been proposed for various nutrients, including the amino acid tryptophan (Trp) and its catabolite 5-hydroxytryptamine (serotonin, 5-HT) [4,5], and also for several enzymes in Trp metabolism. A loss-of-function mutation (G1463A) in *tryptophan hydroxylase-2 *(*TPH2*), for example, the rate-limiting enzyme in 5-HT synthesis ([6]; Figure 1A), has been identified as a factor in unipolar major depression [7]. Further, antidepressants have been postulated to act by directly inhibiting the activity of tryptophan 2,3-dioxygenase (TDO/tryptophan pyrolase) [8-10], one of two rate-limiting enzymes for the kynurenine pathway of Trp metabolism (Figure 1A), in turn enhancing the availability of cerebral Trp [11,12]. In several genetic analyses, human TDO2 gene polymorphisms have been potentially associated with psychiatric diseases, such as Tourette syndrome, depression, and autism [13,14]. Conflicting results have also been reported [15], however, and a direct molecular link between amino acid metabolism and mental disorders/affective status has not been well established.

Here, to better understand the metabolic pathways and enzymes responsible for anxiety-related behavior, we generated *Tdo *knock-out (*Tdo*^-/-^) mice and assessed the role of TDO in anxiety-related behavior and neurogenesis, and in systemic and brain Trp metabolism.

## Results

### Generation of mice with targeted disruption of the *tdo *gene locus

We first disrupted the *tdo *gene in mice by homologous recombination. The targeting vector was constructed by replacing genomic *tdo *exons 1 and 2 (containing the translational initiation site) with the PGK-neomycin (Neo) cassette (Figure 1B). Heterozygous mice were crossed with C57BL/6 mice for five generations. Interbreeding of the resultant heterozygotes produced wild-type (*Tdo*^+/+^), heterozygote (*Tdo*^+/-^), and homozygote (*Tdo*^-/-^) mice, as identified by Southern blot analysis of *Pvu*II-digested genomic tail DNA (Figure 1C). The disruption of *tdo *was verified by the absence of *tdo *mRNA transcripts and TDO protein in the liver, as assessed by quantitative real-time PCR and Western blot analyses, respectively (Figure 1D and 1E). The null mutation of *tdo *was also verified by enzyme activity assays of liver extracts (Figure 1F). These mutant mice were born at ratios that followed Mendelian inheritance and matured for at least one year without apparent gross abnormalities. As TDO is predominantly expressed in the liver, we briefly looked at gross liver morphology: although little difference between 13-week-old *Tdo*^-/- ^and *Tdo*^+/+ ^mice was seen, confirmation of a lack of effect on the histological status of the liver will require further detailed examination.

### Marked increase in plasma Trp and altered plasma Trp metabolite levels in *Tdo*^-/- ^adult mice

**Figure 2:**
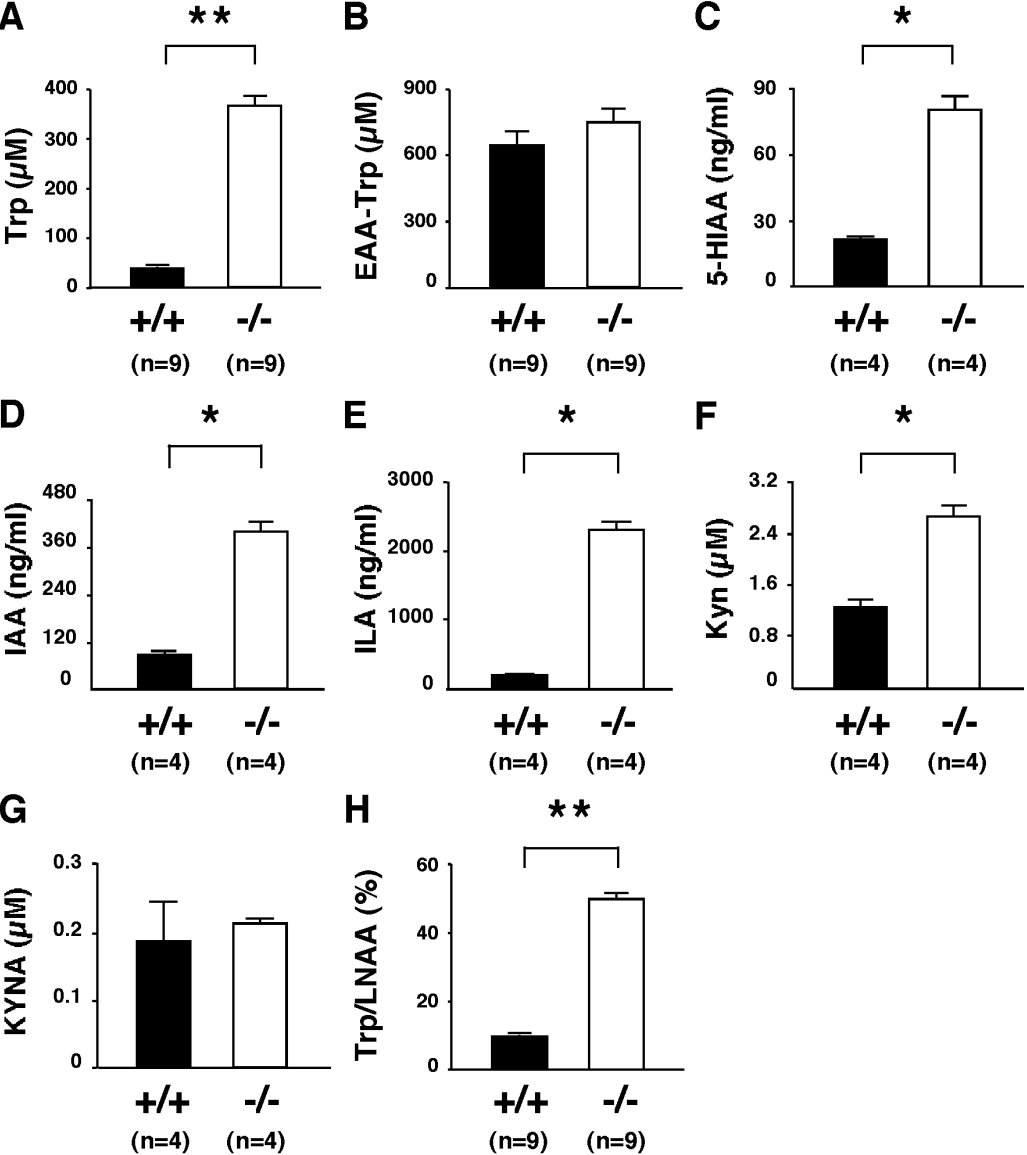
**Effect of *tdo *deletion on systemic Trp metabolites**. (A-H) Plasma amino acid composition and Trp metabolites in 18- to 20-week-old *Tdo*^+/+ ^(+/+) and *Tdo*^-/- ^(-/-) mice. Plasma Trp concentration (A), other essential amino acid (EAA-Trp) concentrations (B), and the ratio of Trp to large neutral amino acids (LNAA, H) were determined using an amino acid analyzer (means ± S.E.). **, *p *< 0.0001. Plasma levels of 5-HIAA (C), IAA (D), ILA (E), kynurenine (Kyn: F), kynurenic acid (KYNA: G) were determined using HPLC-FD and HPLC-UV systems. Values represent means ± S.E. *, *p *< 0.05.

Since TDO is an early and rate-limiting enzyme for Trp metabolism (Figure 1A) and may control systemic Trp metabolism, we first examined the contribution of the *tdo *null mutation to systemic levels of Trp and its metabolites/catabolites. Results showed that Trp levels in plasma were 9.3-fold higher in *Tdo*^-/- ^than *Tdo*^+/+ ^mice (Figure 2A and additional file 1, Table S1). In contrast, no obvious differences were seen in the levels of other essential amino acids (EAAs), except for modest increases in threonine (1.3-fold) and methionine (1.3-fold) (Figure 2B and additional file 1, Table S1). Measurement of the three Trp catabolites in plasma by HPLC showed much higher levels of 5-HIAA, the catabolic product of Trp via the serotonin (5-HT) pathway (Figure 1A(b)), in *Tdo*^-/- ^than *Tdo*^+/+ ^mice (Figure 2C), as well as increases in indoleacetic acid (IAA) and indolelactic acid (ILA) (Figure 2D and 2E), products of Trp via the transamination pathway (Figure 1A(c)). In contrast, no difference was seen in the plasma levels of Trp between mice with knock-out of *indoleamine 2,3-dioxygenase *(*IDO*) [16], one of the two enzymes that convert Trp to formylkynurenine ([17,18]; Figure 1A), and wild-type littermates (see additional file 1, Figure S1). These findings clearly demonstrated that TDO is indeed largely responsible for the systemic level of Trp, and that TDO contributes to Trp catabolites (5-HIAA, ILA, and IAA) levels in plasma, even in the presence of IDO.

We then examined whether levels of the major Trp metabolites kynurenine (Kyn) and kynurenic acid (KYNA) were reduced by *tdo *deletion, on the basis that the marked elevation of Trp in *Tdo*^-/- ^mice suggested the insufficient compensatory conversion of Trp to formylkynurenine by IDO. Kyn levels were 2-fold higher in *Tdo*^-/- ^than *Tdo*^+/+ ^mice (Figure 2F), whereas KYNA levels showed no significant difference (Figure 2G).

### Enhanced brain 5-HT synthesis in adult *Tdo*^-/- ^mice

**Figure 3:**
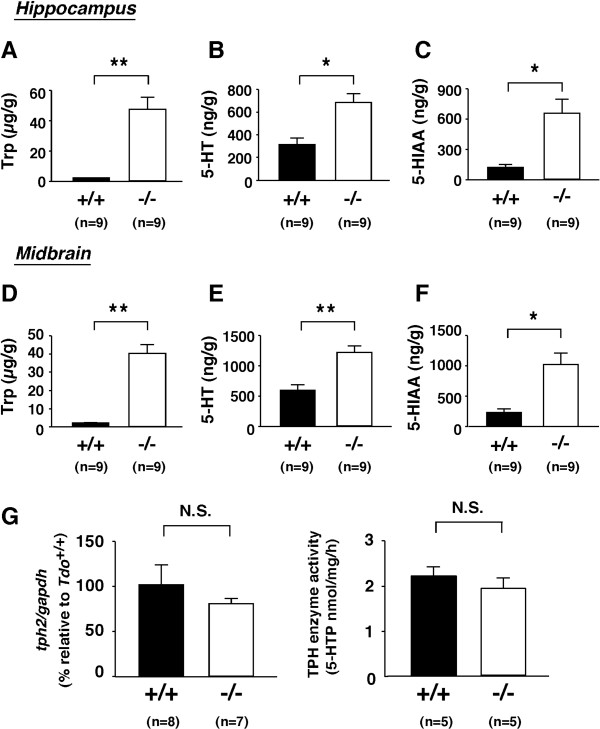
**Modulation of brain Trp and serotonin metabolism by *tdo *disruption**. (A-F) Trp and serotonin (5-HT) metabolites of the hippocampus (A-C) and midbrain (D-F) in 18- to 20-week-old *Tdo*^+/+ ^(+/+) and *Tdo*^-/- ^(-/-) mice. Trp (A, D), 5-HT (B, E), and 5-HIAA (C, F) contents in the hippocampus (*upper*) and midbrain (*middle*) were determined using an HPLC-FD system (means ± S.E.). *, *p *< 0.01. **, *p *< 0.0001. (G) *tryptophan hydroxylase *(*tph2*) mRNA levels and enzymatic activity in the adult midbrain of *Tdo*^+/+ ^(+/+) and *Tdo*^-/- ^(-/-) mice. (*left*) *tph2 *mRNA expression in the midbrain. TaqMan RT-PCR analyses were performed using total RNA extracted from the midbrain of 14- to 16-week-old mice. After normalization to *gapdh*, data were expressed as % fluorescent units relative to those in *Tdo*^+/+ ^mice. Data represent means ± S.E. (*right*) Assay for TPH enzymatic activity. TPH enzyme activity was measured using midbrain homogenates obtained from 15-week-old mice of each genotype. Values represent means ± S.E. N.S., not significant.

Given that large neutral amino acids (LNAA) compete for transport across the blood-brain barrier [2], the elevation of Trp only among LNAA and consequent increase in the Trp/LNAA ratio (Figure 2H) in *Tdo*^-/- ^mice suggested the increased transport of Trp across the blood-brain-barrier. We therefore predicted that 5-HT synthesis in the brain would be altered in *Tdo*^-/- ^mice [19]. On HPLC analysis, Trp concentrations in the hippocampus and midbrain were more than 20-fold higher in *Tdo*^-/- ^than *Tdo*^+/+ ^mice (Figure 3A and 3D), versus no obvious differences for the other amino acids, except methionine (see additional file 1, Table S2). Levels of 5-HT in the midbrain (region of 5-HT synthesis) and hippocampus (region of serotonergic input) were also nearly 2-fold higher in *Tdo*^-/- ^than *Tdo*^+/+ ^mice (Figure 3E and 3B), and those of the 5-HT metabolite 5-HIAA were nearly 5-fold higher (Figure 3C and 3F), indicating the acceleration of 5-HT synthesis and turnover in these mice. Given the notion that tryptophan hydroxylase (TPH) is the rate-limiting enzyme for 5-HT biosynthesis, we asked whether the level or activity of *tph2 *would be modulated by TDO deletion. As shown in Figure 3G, however, mRNA level and enzymatic activity for TPH were not modified in *Tdo*^-/- ^mice, indicating that TDO, rather than TPH, was the dominant regulator of 5-HT biosynthesis under physiological conditions *in vivo*.

### Modulation of anxiety-related behavior in 13- to 15-week-old *Tdo*^-/- ^mice

We next assessed whether TDO deletion modulated anxiety-related behavior. There were a number of rationales for this: changes in levels of Trp and its metabolite 5-HT modulate either or both anxiety- and depression-related behavior [20,21]; the important contribution proposed for Trp and 5-HT dysregulation in many psychiatric disorders, including anxiety, depression, schizophrenia, aggression, autism and alcoholism [20]; the possibility that TDO is directly involved in several psychiatric conditions, as evidenced by the potential association of human TDO2 gene polymorphisms with psychiatric diseases, such as Tourette syndrome, depression, and autism [13,14]; and the possibility that familial hypertryptophanemia, with its associated mood abnormalities, is due to an inborn error in the normal conversion of Trp to Kyn, albeit that the causal gene for this disease has not yet been identified [22]. Results showed that brain levels of Trp and 5-HT [2] were markedly elevated in *Tdo*^-/- ^mice (Figure 3).

**Figure 4:**
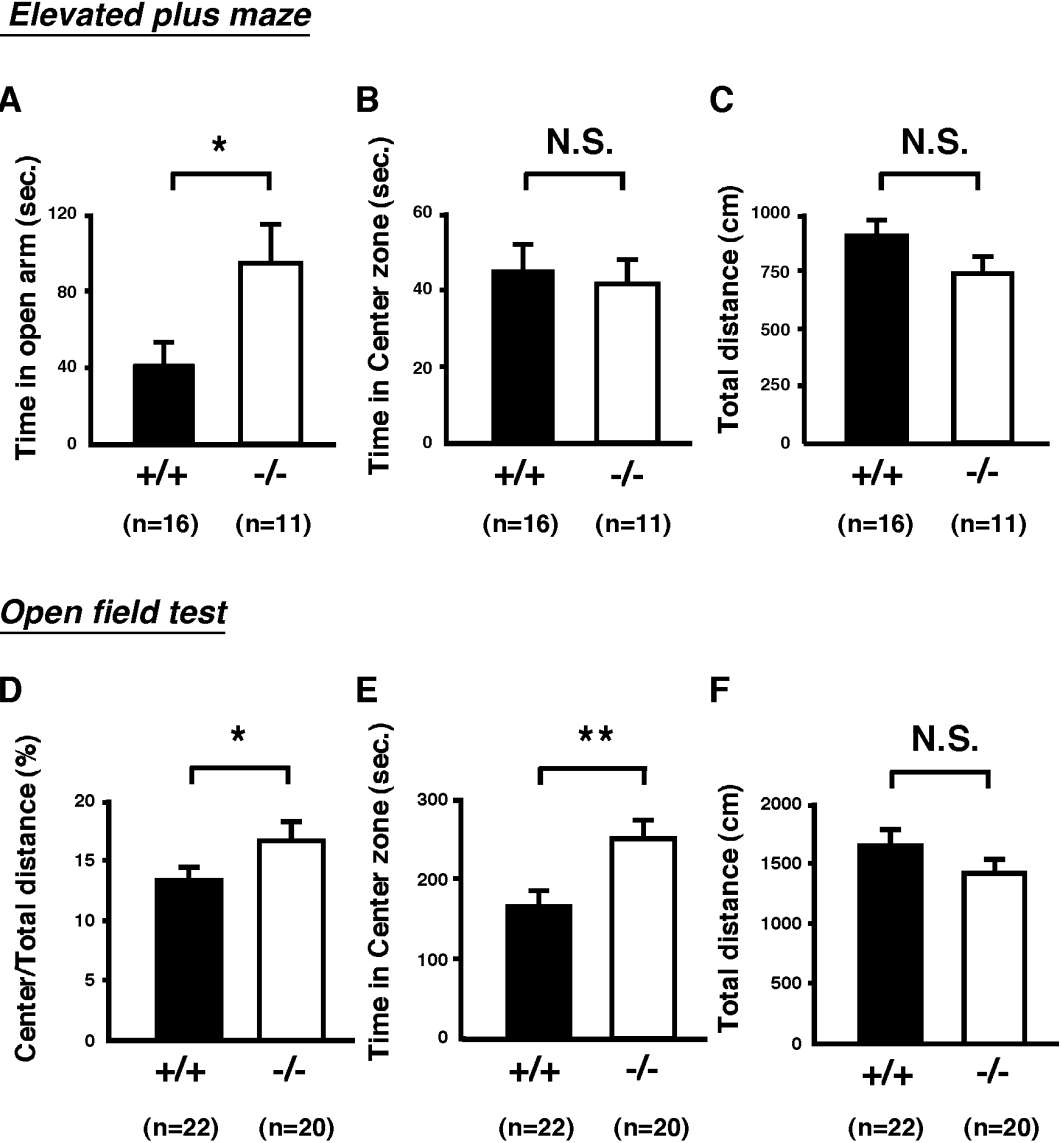
**Anxiety-related behavior tests of 13- to 15-week-old *Tdo*^+/+ ^(+/+) and *Tdo*^-/- ^(-/-) mice**. (A-C) Elevated plus maze tests. Duration in the open arms (A) and center zone (B), and total distance traveled (C) were scored for 5 min. Data represent the mean ± S.E. (D -F) Open field tests. The ratio between locomotion in the center and total locomotion (D), duration in the center zone (E), and total distance traveled (F) were scored for 30 min. Data represent the mean ± S.E. *, *p *< 0.05. **, *p *< 0.01. N.S., not significant.

To clarify the role of TDO deletion in anxiety-related behavior, we next conducted two traditional anxiety-behavior analyses in 13- to 15-week-old mice, the elevated plus maze test (EPM) and the open field test (OFT). In the EPM, *Tdo*^-/- ^mice spent significantly longer times in the open arms of the maze than *Tdo*^+/+ ^mice (Figure 4A). In contrast, no obvious differences were seen in locomotor activity (Figure 4C) or time spent in the center zone (Figure 4B). In the OFT, moreover, *Tdo*^-/- ^mice showed increased center locomotion (Figure 4D) and time spent in the center zone (Figure 4E), but no difference in total movement in the open field (Figure 4F). These results clearly indicate that depletion of TDO induced anxiolytic effects without affecting locomotor activity or the behavioral phenotype of *Tdo*^-/- ^mice.

### Increased proliferation of neural progenitors in the dentate gyrus of the hippocampus in 13-week-old *Tdo*^-/- ^mice

**Figure 5:**
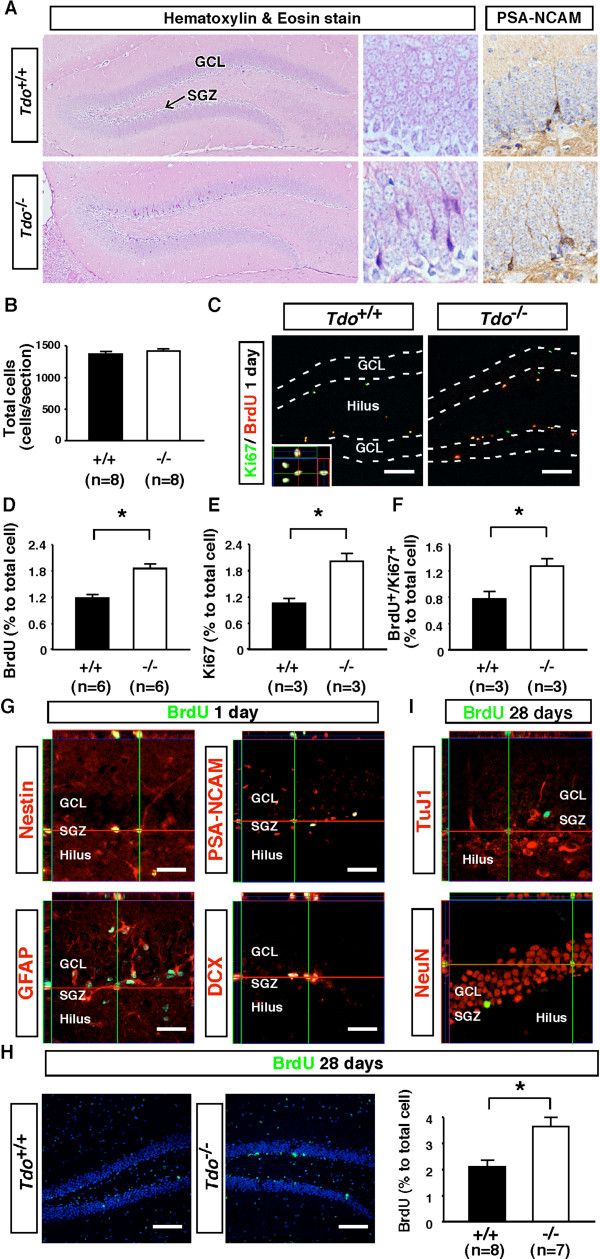
**Increase in neurogenesis in the subgranular zone (SGZ) following *tdo *deletion**. (A) H&E staining of the dentate gyrus (DG) of paraffin-embedded coronal brain sections (5 μm) of 13-week-old *Tdo*^+/+ ^and *Tdo*^-/- ^mice. *Center and right panels*, higher magnification views. *Right panels*, PSA-NCAM-immunostaining. (B-I) Estimation of proliferating neural precursors and neurogenesis in the hippocampus of 13-week-old *Tdo*^+/+ ^and *Tdo*^-/- ^mice. (B) Total number of nuclei/section in the SGZ and granular cell layer (GCL) is shown as total cells. (C) BrdU/Ki67-double staining in the DG at 24 h after BrdU injection. (D-F) BrdU-, Ki67-, and double-labeled cells are indicated as a percentage of total cells. (G) Double-immunostaining of anti-BrdU and neural markers (Nestin, GFAP, PSA-NCAM, or DCX) in the SGZ of *Tdo*^-/- ^mice at 24 h after BrdU injection. (H) BrdU-immunostaining in the DG at 28 days after BrdU injection. Nuclei were stained with TO-PRO-3 (*blue*). (I) TuJ1 and NeuN were co-labeled with anti-BrdU at 28 days after BrdU injection. Orthogonal images show three dimensional analyses of individual cells marked by intersecting lines in the *x*, *y*, and *z *axes. Bars: 100 μm (C, H) and 30 μm (G, I). Data represent means ± S.E. *, *p *< 0.05 versus *Tdo*^+/+ ^mice.

Given the previous finding that X-irradiation of a restricted region of the mouse brain containing the hippocampus inhibited the neurogenic and anxiety-related behavioral effects of two classes of antidepressants, suggesting that the behavioral effects of chronic antidepressant use were mediated by the stimulation of neurogenesis in the hippocampus [23], we next examined whether neurogenesis is accelerated in the adult hippocampus of *Tdo*^-/- ^mice. On H&E staining, hippocampus of 13-week-old *Tdo*^-/- ^mice showed a greater number of deeply stained cells with neurite-like spines in the subgranular zone (SGZ) and granular cell layer (GCL) (Figure 5A, left panel). Some of these cells were immunopositive for PSA-NCAM, a marker for migrating neuroblasts (Figure 5A, right panel). These cells were then characterized by injecting the mice with 5-bromo-2'-deoxyuridine (BrdU; 4 × 75 mg/kg) and sacrificing them 24 h later to assess the number of progenitors that had incorporated BrdU. Although no significant difference among genotypes was seen in the total number of cells in the SGZ and GCL (Figure 5B), the total number of BrdU-labeled cells was markedly higher in *Tdo*^-/- ^than *Tdo*^+/+ ^mice (Figure 5C and 5D), suggesting enhanced proliferation and raising the concern that there is different survival rate of new born cells between wild-type and *Tdo*^-/- ^mice. Confirming these BrdU results, more than 87% of BrdU-positive cells were co-labeled with Ki67, another marker for proliferating cells (Figure 5C). Quantitatively, Ki67-positive proliferating cells in the hippocampal SGZ and GCL were increased 1.9-fold, BrdU-positive cells 1.58-fold, and Ki67/BrdU double-positive proliferating cells 1.64-fold in *Tdo*^-/- ^over *Tdo*^+/+ ^mice (Figure 5C–5F).

To more precisely define the population of BrdU-positive cells, *Tdo*^-/- ^and *Tdo*^+/+ ^sections were double-stained with BrdU and nestin, a neural stem cell marker; the neural progenitor (migrating neuroblast) markers DCX and PSA-NCAM; and GFAP, on the basis of the recent identification of GFAP-positive cells as neural progenitor cells in the adult forebrain and hippocampus [24]. Results showed a significant increase in BrdU-positive cells co-labeled with nestin, GFAP, DCX, or PSA-NCAM in the SGZ of *Tdo*^-/- ^mice, indicating the promotion of both neural stem and progenitor cell proliferation in the SGZ of these mice (Figure 5G and Table 1).

**Table 1:** Quantitative analysis of proliferating neural progenitors in SGZ 24 h after BrdU injection.

	Nestin ^+^	GFAP ^+^	PSA-NCAM ^+^	DCX ^+^
*Tdo *^+/+^	2.78 ± 0.23	5.31 ± 0.49	4.20 ± 0.45	0.80 ± 0.08
*Tdo *^-/-^	2.38 ± 0.13	6.90 ± 0.26*	6.07 ± 0.48*	1.49 ± 0.13*

				

	Nestin^+^/BrdU^+^	GFAP^+^/BrdU^+^	PSA^+^/BrdU^+^	DCX^+^/BrdU^+^

*Tdo *^+/+^	0.58 ± 0.12	0.34 ± 0.06	0.42 ± 0.08	0.77 ± 0.12
*Tdo *^-/-^	1.15 ± 0.13*	0.47 ± 0.05*	0.93 ± 0.09*	1.27 ± 0.11*

Cells positive for neural progenitor markers, alone and in combination with BrdU, are indicated as a percentage of total cells. Cells were counted in >3 matched sections from the DG of each mouse (*n *= 3 for each genotype). These data represent means ± S.E. *, *p *< 0.05 versus *Tdo*^+/+ ^mice (ANOVA, Scheffe's post-hoc test).

Neural progenitors of the SGZ mature locally into granule neurons of the dentate gyrus (DG), sending axonal projections to the CA3 area and dendritic arbors into the molecular layer of the hippocampus. To assess the fate of BrdU-positive neural stem and progenitor cells, mice were sacrificed 28 days after BrdU injection. A large increase in the number of BrdU-positive cells was observed throughout the GCL and outer DG regions in *Tdo*^-/- ^mice (Figure 5H). By degree of differentiation, co-labeling of these BrdU-incorporated cells with TuJ1, an early neuronal marker, and NeuN, a marker of differentiated neurons, showed a 1.6-fold and 2.4-fold increase in early versus differentiated neurons, respectively (Figure 5I and Table 2). Interestingly, these increases were greater than those observed following anti-depressant injection [23], suggesting a marked acceleration of *de novo *neurogenesis in *Tdo*^-/- ^mice.

**Table 2:** Quantitative analysis of adult neurogenesis in SGZ 28 days after BrdU injection.

	NeuN ^+^	TuJ1 ^+^	NeuN^+^/BrdU^+^	TuJ1^+^/BrdU^+^
*Tdo *^+/+^	91.28 ± 1.30	2.75 ± 0.33	0.47 ± 0.12	0.64 ± 0.12
*Tdo *^-/-^	89.62 ± 0.87	5.16 ± 0.47*	1.14 ± 0.12*	1.00 ± 0.13*

Cells positive for neural markers, alone and in combination with BrdU, are indicated as a percentage of total cells. Cells were counted in >3 matched sections from the DG of each mouse (*n *= 3 for each genotype). These data represent means ± S.E. *, *p *< 0.05 versus *Tdo*^+/+ ^mice (ANOVA, Scheffe's post-hoc test).

### Increased proliferation of neural progenitors in the subventricular zone and marked reduction in size of the lateral ventricles in 13-week-old *Tdo*^-/- ^mice

**Figure 6:**
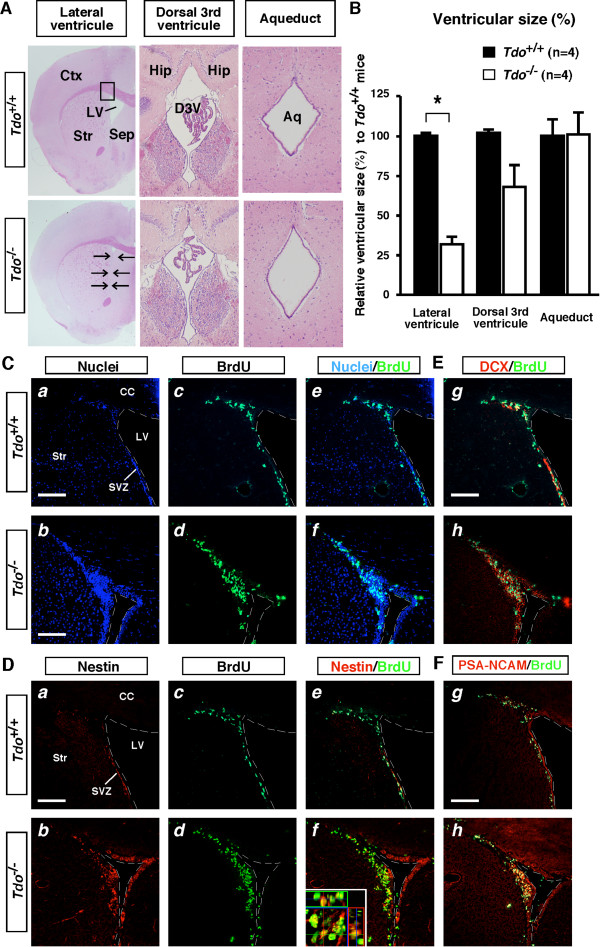
**Reduction in LV size and increased neural progenitors proliferation in the SVZ of *Tdo*^-/- ^mice**. (A) Paraffin-embedded coronal brain sections (5 μm) were stained with H&E, and the size of each ventricle was measured. The boxed area in the subventricular zone (SVZ) was used for studies in Figures 6C-F. Arrows illustrate the change in the size of the LV in *Tdo*^-/- ^mice. (B) Quantitative size of each ventricle in both genotypes. Relative mean size in each ventricle of *Tdo*^+/+ ^mice was defined as 100%. Results are expressed as the means ± S.E. and tested for significance with ANOVA and Scheffe's post hoc test (*p *< 0.05). (C-F) Incorporation of BrdU in neural progenitors of SVZ of *Tdo*^+/+ ^and *Tdo*^-/- ^mice. Frozen coronal sections (20 μm) of the SVZ of the brain of 13-week-old mice were stained with TO-PRO-3 (nuclei, Ca and Cb), BrdU (Cc-f, Dc-f, E, and F), DCX (E), PSA-NCAM (F), nestin (Da, Db, De and Df) (boxed area in Figure 6A) at 24 h after BrdU injection. Inset, a merged view at high magnification (Df) of BrdU (*green*) and nestin (*red*). Ctx, cortex; Str, striatum; Sep, septum; Hip, hippocampus; D3V, dorsal 3rd ventricle; Aq, Aqueduct; and CC, corpus callosum. Bars: 100 μm.

To clarify whether this acceleration of neurogenesis was restricted to the hippocampus, we next examined neurogenesis in the subventricular zone (SVZ), the second region showing adult neurogenesis in mammals. On H&E staining of coronal sections, the lateral ventricles (LVs) of 13-week-old *Tdo*^-/- ^mice brains were only one-third the size of those of *Tdo*^+/+ ^mice. In contrast, no difference was seen in other ventricular regions, including the dorsal third ventricle (D3V) and aqueduct (Aq) (Figure 6A and 6B). Given that the subventricular zone (SVZ) is situated throughout the lateral wall of the LV and is one of only two regions that participate in adult neurogenesis in mammals, and that higher levels of either or both Trp and 5-HT may thus affect morphological changes via the acceleration of neurogenesis, we next assessed the modification of neurogenesis in SVZ of *Tdo*^-/- ^mice.

To explore this possibility, BrdU (4 × 75 mg/kg) was injected into 13-week-old mice, which were then examined 24 h later. On TO-PRO-3 iodide (nuclear) staining, markedly more cells were seen in the SVZ of *Tdo*^-/- ^than *Tdo*^+/+ ^mice, particularly in the region surrounded by corpus callous (CC), striatum (Str) and LV, (Figure 6Ca and 6Cb). Further, *Tdo*^-/- ^mice also showed more BrdU-positive cells in this region (Figure 6Cc–f) than wild-type mice. Immunostaining for nestin (red) or nestin plus BrdU (green, overlay view) in *Tdo*^-/- ^mice revealed noticeably more nestin-positive neural stem cells in the SVZ, not only on the Str but also on the CC side (Figure 6D), suggesting marked neural stem cell proliferation in the SVZ. With regard to neural progenitor (migrating neuroblast) cells, immunostaining for DCX (red) and BrdU (green, overlay view) revealed a perceptible increase in the number of proliferating DCX-positive neural progenitor cells in the SVZ of *Tdo*^-/- ^mice (Figure 6E). Similar results were obtained using PSA-NCAM as an additional marker for neural progenitor cells (Figure 6F). Taken together, these findings demonstrate that the loss of TDO induces the proliferation of both neural progenitors and neural stem cells in the SVZ, and hence might contribute, either fully or partly, to a decrease in the size of the LV.

### Accelerated adult neurogenesis in the GCL of the olfactory bulb in 13-week-old *Tdo*^-/- ^mice

Adult-born cells in the SVZ migrate along the rostral migratory stream to the olfactory bulb (OB) where they differentiate into interneurons. To determine if the number of newly generated neurons migrating to their final destination in the OB was altered in *Tdo*^-/- ^mice, BrdU was administered to 9-week-old mice, which were sacrificed 4 weeks later. Although Cresyl violet-stained brain sections showed little difference between genotypes in the appearance of the OB (see additional file 1, Figure S2A), the estimated number of BrdU-positive cells in the GCL of the OB was 1.6-fold higher in *Tdo*^-/- ^than *Tdo*^+/+ ^mice (see additional file 1, Figure S2C and S2E, middle panels). These mice also showed an increase in the number of BrdU/PSA-NCAM double-positive proliferating neuroblasts, presumably derived from the SVZ (see additional file 1, Figure S2B and S2C, right panels); and a notable increase in the number of BrdU-positive cells co-labeled with NeuN in the GCL (see additional file 1, Figure S2D and S2E, right panel). These findings indicate that the enhanced proliferation of neural progenitors in the SVZ increased the number of migrating neuroblasts and enhanced adult neurogenesis in the GCL in the OB of 13-week-old *Tdo*^-/- ^mice. Total PSA-NCAM- and NeuN-positive cell numbers of olfactory bulb were not altered in *Tdo*^-/- ^mice in a similar manner with dentate gyrus, raising the concern that there is different survival rate of new born neural progenitors and neurons between wild type and *Tdo*^-/- ^mice.

## Discussion

Using mice deficient for *tdo*, we provide the first evidence that TDO, one of two initial and rate-limiting enzymes for the kynurenine pathway of Trp metabolism, is directly linked to systemic Trp metabolism, neurogenesis and anxiety-related behavior *in vivo*. These mice had markedly increased plasma levels of Trp, 5-HIAA, ILA and IAA in the presence of IDO, demonstrating that the accumulation of Trp and acceleration of serotonergic and transamination pathways are largely dependent on TDO (Figure 1 and 2). Although other tryptophan metabolic enzymes, such as kynurenine formamidase, may also play a role in the regulation of systemic Trp levels and several important neural functions [16,25-27], we propose that the greater severity of biochemical changes in our *Tdo*^-/- ^mice, particularly in systemic Trp levels, than in other animal models indicates that TDO is the key regulatory enzyme in the modulation of systemic Trp levels. In addition to Trp, 5-HT and 5-HIAA levels were also elevated in the hippocampus and midbrain of these mice. Taken together, these findings demonstrate that TDO, which is expressed predominantly in the liver, plays an essential and dominant role in the *in vivo *regulation of brain levels of 5-HT, even in the presence of tryptophan hydroxylase-1 (in the periphery) and tryptophan hydroxylase-2 (in the brain). This finding contrasts with those of previous studies, which have indicated the latter two enzymes as rate-limiting for 5-HT synthesis [6,28].

In contrast to our findings for Trp, plasma levels of Kyn and KYNA, which are downstream Trp metabolites generated by TDO (Figure 1), were sustained at physiological levels despite the absence of TDO. This finding suggests the presence of compensatory mechanisms to maintain Kyn and KYNA levels in *Tdo*^-/- ^mice; and given that IDO mediates the same metabolic processes as TDO in various tissues, it is the most likely candidate. On this basis, IDO would be expected to decrease plasma and brain Trp levels but increase its downstream metabolites Kyn and KYNA [29]. Our assessment of both TDO and IDO enzyme activities from liver lysates, however, showed a loss in the conversion of Trp to Kyn in *Tdo*^-/- ^compared with *Tdo*^+/+ ^mice (Figure 1F), suggesting that the compensatory mechanism(s) may function in extra-hepatic tissues and, in part, play a role in decreasing Trp level and increasing Kyn level. In addition, a modulatory mechanism(s) in Kyn pathway may also play a role. Increasing Trp concentrations in food decrease the enzymatic activity of quinolinate phosphoribosyltransferase (QPRT), a downstream metabolic enzyme of TDO [30]. Although the mechanism remains unclear, our findings thus raise the possibility that Trp metabolism downstream of Kyn and KYNA plays a role in maintaining plasma Kyn and KYNA levels in *Tdo*^-/- ^mice.

We also used these *Tdo*^-/- ^mice to evaluate the role of TDO in anxiety-related behavior. Although the mechanism remains to be elucidated, TDO deletion had clear anxiolytic effects, as revealed by two classical behavioral tests. In agreement with our data, Yamasaki et al. reported marked reduction of the level of *tdo *mRNA in the hippocampus of alpha-CaMKII deficient mice (alpha-CaMKII^+/-^) that show anxiolytic phenotype [31]. Trp and its catabolite 5-HT are thought to modulate mood control [4]. Given that the roles of 5-HT1A receptor and 5-HT transporter during development in anxiety-related behavior have been reported, respectively [32,33], it is postulated that *Tdo*^-/- ^mice show anxiolytic change due to 5-HT-upregulation in the brain and subsequent modulation of neural development. In addition, in the adult, given postulation that 5-HT/5-HT_1A _receptor-mediated neurogenesis is critically involved in the anxiolytic effects of anti-depressant fluoxetine [23], one likely mechanism of this is that the deletion of hepatic TDO modulates plasma Trp and subsequently increases brain Trp and 5-HT, which in turn accelerates neurogenesis in the hippocampus. It should be noted that *tdo *and its variants mRNAs are expressed in various regions of developing and adult brain [34], suggesting a possible role of locally expressed TDOs in the brain for the specific regional modulation of the brain and subsequent behavioral modulation. Moreover, altered immunoreactivity against TDO has been reported in patients with schizophrenia and depression [35]. In addition, given findings of a correlation between Kyn levels and the regulation of behavior in insects and of an increase in plasma Kyn concentration in endogenous anxiety in humans, we cannot exclude the possibility that anxiety-related behavior is also modulated by TDO-induced changes in Kyn, and possibly in other kynurenines as well [36,37]. If the contribution of alterations in Kyn to anxiety-related behavior is indeed important, then TDO would appear to be a key modulator of this behavior under physiological conditions via the control of both 5-HT and Kyn. This possible role of TDO stands in contrast to that of TPH, which has been considered a rate-limiting enzyme in the synthesis of 5-HT but not of Kyn.

The role of stress and stress-induced glucocorticoids in affecting mood and anxiety is well known. Administration of glucocorticoids to rats results in elevations of the tryptophan-metaboliting enzymes and TPH *in vivo *and that administration of dexamethasone phosphate regulates TDO activity in cells from control and adrenarectomized mice, respectively [38,39]. In addition, glucocorticoids regulate either or both the activity and mRNA levels of TDO in rat liver [40] and isolated primary hepatocytes [41,42]. Indeed, stresses such as forced running, immobilization and exposure to cold increase rat liver TDO activity [43]. Taken together, our findings raise the possibility that TDO may in part contribute to the modulation of mood and anxiety-related behavior by stress and environment (see additional file 1, Hypothetical model in Figure S3).

## Conclusion

In summary, we provide the first evidence that TDO plays an essential role in the homeostasis of systemic and brain Trp metabolism, including the dominant regulation of serotonergic pathway, under the physiological conditions. TDO also play a role in the maintenance of brain morphology via regulating adult neurogenesis in the hippocampus and subventricular zone. Furthermore, TDO modulates anxiety-related behavior, indicating a role of TDO in higher brain functions. Collectively, the present findings in *Tdo*^-/- ^mice indicate a direct molecular link between tryptophan metabolism and mental status. *Tdo*^-/- ^mice will likely prove useful in clarifying the physiological role of Trp metabolism in normal brain function and in psychiatric disorders, and for development of new approaches for therapeutic interventions of mental disorders.

## Methods

### Experimental animals

Mice were housed in groups of 3–4 per cage in a room with controlled light (12 h light/dark cycle; lights on at 9 A.M.), humidity, and temperature, and allowed *ad libitum *access to food and water. Only males were used for the analyses. The acquisition, care, housing, use, and disposition of the animals were in compliance with the institutional laws and regulations of the Osaka University Graduate School of Medicine. All efforts were made to minimize animal discomfort and the number of animals used.

### Construction of the targeting vector

Genomic DNA clones of the *tdo *locus were obtained from the 129/SvJ mouse-derived genomic library ([44]; the kind gift of Dr. T. Morita, Osaka University) using rat *tdo *cDNA [10] as a probe. Among the clones, a 12.5-kb *tdo *genomic fragment containing exons 1 to 3 was used to construct a *tdo *targeting vector, which was prepared by replacing exons 1 and 2 of the *tdo *fragment containing the translation initiation site with the PGK-neomycin (Neo) cassette at *Hind*III-*Xba*I sites. Subsequently, the *Apa*I-*Eco*RI 9.0-kb fragment (left arm, 6.5-kb; right arm, 2.5-kb) was excised and inserted into the MC1 promoter driven diphtheria toxin (DT)-A cassette in order to connect the 5' end of the insert to DT-A.

### Disruption of the *tdo *locus

R1 embryonic stem (ES) cells (kindly provided by Dr. A. Nagy, Mt. Sinai Hospital, Canada, via Dr. H. Kondo, Osaka University, Japan) were electroporated with linearized targeting vector DNA, and selected with G418. G418-resistant ES clones harboring the desired homologous recombinations were verified by Southern blot analysis as previously described [44,45] after the genomic DNA was digested with *Pvu*II, using a probe specific for the intron sequence between exons 3 and 4 of *tdo *(Figure 1B). ES cells that underwent homologous recombination were microinjected into(C57BL/6 × DBA/2) F1 (BDF1) blastocytes. Male chimeras were crossed with C57BL/6 females to generate germ-line heterozygous offspring, with transmission of the targeted allele verified by Southern blot analysis. After backcrossing with wild-type C57BL/6 mice (SLC, Shizuoka, Japan) for 5 generations, homozygous *tdo *mutants and wild-type animals were obtained by intercrossing heterozygotes. Genotyping of progeny was performed by Southern blot analysis of tail-derived genomic DNA.

### RNA purification and quantitative real-time RT-PCR

Total RNA was purified from the livers of 15-week-old wild-type (*Tdo*^+/+^), heterozygote (*Tdo*^+/-^), and homozygote (*Tdo*^-/-^) mice using a TRIzol reagent (Invitrogen) according to the manufacturer's instructions. Quantitative real-time RT-PCR was carried out and mRNA levels were calculated as described previously [46]. For amplification of mouse *tdo, tph2*, and *gapdh *(the endogenous control), Universal PCR master mix and FAM dye-labeled Taq-Man MGB probes (Applied Biosystems) were used for mouse *tdo *(exons 4 and 5, Mm00431715), *tryptophan hydroxylase-2 (tph2*, Mm00557717_m1) and for rodent *gapdh *(Taq-Man rodent GAPDH control reagents VIC probe), and the results expressed as the mean ± S.E.

### Western blotting

Western blot analyses of liver lysates from 15-week-old *Tdo*^-/-^, *Tdo*^+/-^, and *Tdo*^+/+ ^mice were done using anti-rat TDO antiserum (1:2,000, [47]).

### Assay for TDO activity

Liver homogenates were obtained from 10-week-old *Tdo*^-/-^, *Tdo*^+/-^, and *Tdo*^+/+ ^mice. Assays for hepatic TDO activity were carried out using L-Trp as substrate as previously described [48], with activity expressed as μmol of kynurenine formed per hour per gram of wet liver weight.

### Measurement of amino acids in plasma and brain

Plasma was deproteinated with 5% sulfosalicylic acid, filtered, and immediately analyzed for amino acid concentrations using automated ion-exchange chromatography with lithium-based buffers on a high-speed amino acid analyzer (L8500, Hitachi, Japan), or stored at -80°C. Micro-dissected brain tissues were rapidly removed after perfusion with ice-cold Hanks' balanced salt solution, and extracted in a solution containing 0.5 M HClO_4 _and 0.025% EDTA. The tissue extracts were incubated on ice and centrifuged at 12,000 g at 4°C. The collected supernatants were filtered and amino acid concentrations were determined using the amino acid analyzer (Hitachi).

### Quantitation of Trp metabolites

Plasma samples treated with trichloroacetic acid (TCA) were prepared in the same way as that used for amino acid analysis. Plasma levels of Trp metabolites (Trp, Kyn, KYNA, ILA, IAA, 5-HT, and 5-HIAA) were determined using HPLC-FD and HPLC-UV systems as previously described [49]. The column used for HPLC was a reversed-phase C_18 _(2.0 × 250 mm hypersil BDS column, Hewlett Packard). The Agilent 1100 series fluorescence detector utilized the following excitation and emission wavelengths: 287 nm/340 nm for Trp, ILA, IAA, 5-HT, and 5-HIAA, and 254 nm/404 nm for KYNA. UV signals for Kyn were monitored at 365 nm.

Brain samples were prepared in the same way as those for amino acid analysis. Trp, 5-HT, and 5-HIAA were quantified as previously described [50], with fluorescence detector signals monitored at excitation and emission wavelengths 287 nm/340 nm.

### Assay for TPH activity

TPH activity in fresh brainstem from 15-week-old mice of each genotype was determined as previously described [51] with slight modifications. Assay mixture containing 0.3 mM L-Trp, 0.1 mM Fe(NH_4_)_2_(SO_4_)_2_, 1 mM 6-methyl-tetrahydropterin (6-MPH_4_), 2 mM NSD1015 (a inhibitor of the aromatic amino acid decarboxylase), 25 mM DTT, 2 mg/ml catalase, and 50 mM HEPES, pH 7.2 were reacted at 30°C for 20 min with shaking in the dark. The reaction was terminated by the addition of saturated TCA to a final concentration of 10%, and the mixture was then chilled on ice and centrifuged at 4°C. 5-hydroxytryptophan (5-HTP) in the supernatant was separated on a μBondapak ODS C_18 _HPLC column (Waters) with a mobile phase of 2.5% methanol in 40 mM sodium acetate buffer, pH 3.5. A F1050 fluorescence detector (Hitachi) detected signals at the excitation and emission wavelengths of 287 and 340 nm, respectively. TPH enzymatic activity was normalized to total protein in the homogenate and expressed as nmol 5-HTP/mg/h at 30°C.

### Histological and immunohistochemical analyses

Mice were deeply anesthetized and transcardially perfused with ice-cold phosphate-buffered saline (PBS; pH 7.4), followed by 4% paraformaldehyde (PFA) in PBS. For analyses of brain morphology and PSA-NCAM immunostaining, the brains were removed and embedded in paraffin. Serial coronal sections (5 μm) were prepared, deparaffinized and stained with hematoxylin and eosin (H&E), or used for PSA-NCAM immunostaining. For most immunostaining, brains were removed and cryoprotected in 10% and 20% sucrose in PBS at 4°C after perfusion with 4% PFA in PBS. Twenty-micrometer sections were prepared using a cryostat, mounted on APS -coated slides, and stored at -80°C. Primary antibodies were applied to the sections for 24 or 48 h at 4°C after incubation with blocking buffer containing 10% goat or donkey serum and 0.3% Triton X-100 in PBS. The following primary antibodies were used: Ki67 (DAKO), nestin (BD Pharmingen), GFAP (Chemicon), DCX (Santa Cruz), NeuN (Chemicon), βIII-tubulin (TuJ1; Covance Research), and PSA-NCAM (AbCys S.A.).

After washing with PBS, either Alexa Fluor 488- or Alexa Fluor 546-conjugated secondary antibody and the nuclear counterstaining reagent TO-PRO-3 iodide (Molecular Probes) in PBS were applied. Sections were then washed with PBS, mounted in Crystal mount (Biomeda) and observed under a LSM 510 PASCAL confocal microscope (Zeiss).

### Quantification of ventricular size

The size of each ventricle (lateral ventricle, between 1.1 and 0.86 mm rostral; dorsal 3rd ventricle, between 1.7 and 1.94 mm caudal; and aqueduct, 4.36 and 4.48 mm caudal to the bregma, as per Franklin and Paxinos, [52]) was analyzed in six paraffin-embedded sections per animal (*n *= 4 per group) and quantified using WinRoof software (Mitani Corp., Japan), with the average size of the ventricles in *Tdo*^+/+ ^mice defined as 100%. Results are expressed as the mean ± S.E.

### Administration of BrdU and staining

BrdU (5-bromo-2'-deoxyuridine) injections and subsequent analyses were performed [23] using a BrdU-specific antibody (Oxford Laboratory). In brief, to assess dividing progenitors, 13-week-old mice were administered BrdU (4 × 75 mg/kg) by intraperitoneal injection at 2 h intervals and sacrificed 24 h after the last injection. To determine the fate of BrdU-labeled cells, 9-week-old mice were administered BrdU (4 × 75 mg/kg) and sacrificed 28 days after the last injection. Brain sections were prepared as described above. For BrdU immunohistochemistry, sections were fixed in acetone, treated with 1 N HCl for 30 min at 60°C to denature DNA, and rinsed in PBS. Subsequent processes for immunolabeling with the BrdU antibody were identical to those described above.

### Cell quantitation in the hippocampus

Hippocampal sections from 13-week-old *Tdo*^+/+ ^and *Tdo*^-/- ^mice prepared 1 and 28 days after the injection of BrdU were examined for the number of cells immunopositive for NeuN, TuJ1, PSA-NCAM, DCX, nestin, GFAP, BrdU, and/or Ki67 [23]. In these experiments, cells in the total surface of the granular cell layer (GCL) or in the subgranular zone (SGZ) in the dentate gyrus (DG) were counted in one of at least five sections per animal (*n *> 3 per group) between 1.7 and 2.06 mm caudal to the bregma, as per Franklin and Paxinos [52]. The total number of nuclei/slice in the SGZ and GCL was defined as total cell number. The percentage of double-immunostained cells was obtained by analyzing three-dimensional reconstructed BrdU-positive nuclei in *x-z *and *y-z *orthogonal projections for the presence of cell markers.

### Behavioral assessment using the elevated plus maze test and open field test

All behavioral tests (elevated plus maze and open field test) were conducted between 9:30 AM and 1:00 PM. All experiments were monitored by an automated video camera system and analyzed with Ethovision Ver. 2.3.19 software (Noldus, Wageningen, Netherlands).

#### Elevated plus maze

*Tdo*^+/+ ^and *Tdo*^-/- ^mice (13 to 15 weeks old) were tested in an elevated plus maze according to Lister *et al*. [53] with slight modification. In brief, the plus maze consisted of two open (30 × 6 cm) and two wall-enclosed arms (30 × 6 × 15 cm) connected by a central platform (6 × 6 cm). The apparatus was elevated 40 cm above the floor. The mouse was placed in the central zone facing an open arm, which the animal would usually enter first, and exploratory behavior during a 5 min test period was monitored. Testing took place during the light phase under standard light.

#### Open field test

Open field tests were conducted in 13- to 15-week-old Tdo^+/+ ^and Tdo^-/- ^mice according to the method of Paylor et al. [54]. Briefly, the open field consisted of four adjacent activity chambers (40 × 40 × 40 cm) surrounded by walls, with the field lit from above. Mice were released into the center of the field and allowed to roam the open field for 30 min. Total distance moved, time spent and the distances moved in both the margins (≤10 cm of the walls) and center zone of the field (>10 cm from walls) were measured. The ratio of the distance moved in the center to the total distance moved was calculated and used as a measure of anxiety-related behavior.

### Statistical Analysis

Statistical analysis was carried out using StatView software version 5.0.1 (SAS Institute, Cary, NC). Student's *t*-test was used for the amino acid analyses, while the other data were analyzed by one-way factorial analysis of variance (ANOVA). A post-hoc test was carried out for ANOVA *p*-values less than 0.05. Statistical significance was determined using Scheffe's post-hoc test at *p *< 0.05. Statistical results were indicated as a *p*-value by the post-hoc test.

## Abbreviations

TDO: tryptophan 2,3-dioxygenase; IDO: indoleamine 2,3-dioxygenase; Trp: tryptophan; 5-HT: 5-hydroxytryptamine or serotonin; Kyn: kynurenine; 5-HIAA: 5-hydroxyindoleacetic acid; KYNA: kynurenic acid; ILA: indolelactic acid; IAA: indoleacetic acid; EAA: essential amino acids; LNAA: large neutral amino acids; TPH: tryptophan hydroxylase; EPM: elevated plus maze test; OFT: open field test.

## Competing interests

TN reports that he is employed by Osaka University, which financed development of regenerative medicine by Kringle Pharma Inc. (KP, Toyonaka, Japan).

## Authors' contributions

HF, MK, KM, SM and TN designed the study. HT and TH generated *Tdo*-KO mice. MK selected mice lines of *Tdo*-KO, characterized, backcrossed and provided these mice. MK, HT, TH and HF carried out experiments. HF, MK and TN wrote the manuscript.

## Supplementary Material

Additional file 1 **Supplementary methods, Supplementary Tables S1-S2, and Supplementary Figures S1-S3 are included in this file.** Click here for file
