# Supplementary material for: Tryptophan 2,3-dioxygenase is a key modulator of physiological neurogenesis and anxiety-related behavior in mice
Source: Mol Brain. 2009 Mar 27;2:8. doi: 10.1186/1756-6606-2-8 (PMC2673217; doi:10.1186/1756-6606-2-8)

Additional file

Supplementary Methods

Experimental Animals

Indoleamine 2,3-dioxygenase-deficient (IDO-KO) mice were purchased from the Jackson Laboratory (Bar Harbor, ME), and their offspring were used for experiments. Mouse genotypes were determined by PCR and southern blot analyses as reported previously [16]. C57Bl/6J mice used as controls were purchased from Nippon Clea (Shizuoka, Japan). Mice were housed in groups of 3-4 per cage in a room with controlled light (12h light/dark cycle; lights on at 8 A.M.), humidity, and temperature, and allowed *ad libitum* access to food and water. Only males were used for the analyses. The acquisition, care, housing, use, and disposition of the animals were in compliance with institutional laws and regulations of the Osaka University Graduate School of Medicine. All efforts were made to minimize animal discomfort and the number of animals used.

Cell Quantitation in the Olfactory Bulb

Histological and immunohistochemical analyses are detailed in the text. BrdU-positive cells, cell differentiation markers, and double-labeled cells in 13-week-old *Tdo*$^{+/+}$ and *Tdo*$^{-/-}$ mice were quantitated. In the olfactory bulb (OB), sections from 13-week-old *Tdo*$^{+/+}$ and *Tdo*$^{-/-}$ mice were prepared four weeks after injection of BrdU, and quantitated for the number of cells immunopositive for NeuN, PSA-NCAM, and BrdU

[24]. In these experiments, every ninth section between 4.28 and 3.92 mm rostral to the bregma (n > 3 per group) was analyzed and final cell counts were expressed as cells (in thousands) per mm^3 in the GCL. When the p-value by ANOVA was less than 0.05, Scheffe's post-hoc test was carried out. Statistical significance was indicated as $p < 0.05$ by post-hoc test.

Supplementary Figure Legends

Supplementary Figure S1: Plasma Trp levels in 15-week-old *Tdo*$^{-/-}$ and IDO-deficient Mice

Plasma Trp concentrations in each genotype were determined using an amino acid analyzer (means ± S.E., $n = 4$). No obvious difference was seen in plasma Trp levels between IDO-KO and WT mice, whereas that in *Tdo*$^{-/-}$ mice was markedly increased. These results suggested that TDO, rather than IDO, plays a critical role in regulating systemic Trp metabolism under physiological conditions.

Supplementary Figure S2: Adult Neurogenesis in the Olfactory Bulb (OB) of 13-week-old *Tdo*$^{+/+}$ (+/+) and *Tdo*$^{-/-}$ (-/-) mice

(A) Cresyl violet staining in coronal frozen sections of the OB. (B) Double-staining with PSA-NCAM (red) and BrdU (green) of coronal frozen sections of the OB GCL 28 days after BrdU injection. Inset, a merged view at high magnification of PSA-NCAM and BrdU. (C) Quantification of the average number of PSA-NCAM (left)-, BrdU (middle)-, and PSA-NCAM/BrdU (right)-positive cells in the OB GCL 28 days after

BrdU injection. Data represent means ± S.E. (x10^3 cell/mm^3) from more than nine matched serial sections per mice ($n > 3$ per genotype). **, $p < 0.001$. (D) Double-immunostaining with NeuN (red) and BrdU (green) in coronal frozen sections of the OB granular cell layer (GCL) 28 days after BrdU injection. Inset, a merged view at high magnification of NeuN and BrdU. (E) Quantification of the average numbers of NeuN (left)-, BrdU (middle)-, and NeuN/BrdU (right)-positive cells of the OB GCL 28 days after BrdU injection. Data represent means ± S.E. (x 10^3 cell/mm^3) from more than nine matched, serial sections ($n > 3$ per each genotype). ** $p < 0.001$. Bars: 100 μm (B and D).

Supplementary Figure S3: Model of the Role of TDO in Modulating Anxiety-related Behavior and Neurogenesis through Regulation of Trp Metabolism.

Food intake leads to an increase in the concentration of plasma Trp, which is predominantly metabolized to kynurenine (Kyn) via hepatic TDO, rather than extra-hepatic IDO. Up-regulation of TDO activity in the liver (or locally expressed in the brain) by stress or stress-induced glucocorticoid would cause a decrease in systemic Trp levels, Trp transportation into the brain, and 5-HT biosynthesis in the central nervous system, even in the presence of intact TPH. In contrast, a decrease in TDO activity via factors such as genetic mutation would lead to an increase in systemic and brain Trp/5-HT levels. These changes in Trp metabolism by TDO regulation would modulate anxiety-related behavior and adult neurogenesis, and may be largely involved in the modulation of mood by stress and environment. In addition, changes in the level of Kyn by TDO may also contribute to anxiety-related behavior. We intend to investigate the

role of brain TDO in a future study.

Supplementary Table S1.

Plasma amino acid composition in 18-20-week-old *Tdo*$^{+/+}$ and *Tdo*$^{-/-}$ mice.

	Tdo$^{+/+}$ (n = 9)	*Tdo*$^{-/-}$ (n = 9)	p-value
Tryptophan	38.67 ± 4.66	360.49 ± 15.9	< 0.0001
Valine	158.09 ± 16.0	181.26 ± 11.6	n.s.
Isoleucine	54.28 ± 6.09	55.37 ± 5.16	n.s.
Leucine	95.70 ± 11.3	96.93 ± 8.98	n.s.
Tyrosine	59.63 ± 11.9	50.07 ± 5.56	n.s.
Phenylalanine	64.62 ± 8.74	58.25 ± 3.83	n.s.
Histidine	57.50 ± 4.65	49.07 ± 3.43	n.s.
Aspartic acid	5.93 ± 0.90	4.50 ± 0.47	n.s.
Asparagine	45.91 ± 11.0	30.77 ± 3.17	n.s.
Glutamic acid	30.56 ± 4.80	21.81 ± 2.24	n.s.
Glutamine	889.06 ± 63.5	707.63 ± 64.8	n.s.
Glycine	269.28 ± 26.3	229.70 ± 23.9	n.s.
Threonine	87.06 ± 6.44	117.57 ± 13.4	< 0.05
Serine	93.01 ± 13.3	86.59 ± 7.34	n.s.
Alanine	290.65 ± 44.2	292.65 ± 27.9	n.s.
Cysteine	3.96 ± 1.02	5.89 ± 0.99	n.s.
Methionine	32.52 ± 3.04	43.70 ± 4.28	< 0.05
Arginine	56.58 ± 6.58	60.57 ± 6.13	n.s.
Lysine	162.48 ± 9.29	192.67 ± 12.2	n.s.
Hydroxy-Proline	19.64 ± 2.92	19.78 ± 1.53	n.s.
Proline	68.37 ± 9.53	63.33 ± 7.31	n.s.
Total	2682.7 ± 238.6	2832.6 ± 198.6	n.s.
EAA	693.4 ± 59.2	1106.2 ± 65.8	< 0.005
EAA-tryptophan	654.7 ± 55.6	745.7 ± 51.6	n.s.
LNAA	405.5 ± 51.8	740.5 ± 48.5	< 0.005
Tryptophan/LNAA(%)	9.6 ± 0.5	49.6 ± 1.7	< 0.0001

Plasma amino acid composition in 18-20-week-old *Tdo*$^{-/-}$ mice was determined with an amino acid analyzer. Values represent means ± S. E. (μM). EAA, valine, threonine, tryptophan, methionine, phenylalanine, leucine, isoleucine, and lysine; LNAA, tyrosine, phenylalanine, leucine, isoleucine, tryptophan, and valine; n.s., not significant.

Supplementary Table S2.

Brain amino acid composition in adult *Tdo*$^{+/+}$ and *Tdo*$^{-/-}$ mice.

	Tdo$^{+/+}$ (n = 10)	*Tdo*$^{-/-}$ (n = 11)	p-value
Tryptophan	0.12 ± 0.01	19.57 ± 3.18	< 0.0001
Valine	6.21 ± 0.37	6.47 ± 1.02	n.s.
Isoleucine	2.41 ± 0.14	2.21 ± 0.29	n.s.
Leucine	4.42 ± 0.23	4.21 ± 0.62	n.s.
Tyrosine	3.83 ± 0.40	4.09 ± 0.57	n.s.
Phenylalanine	6.94 ± 1.70	6.84 ± 2.00	n.s.
Histidine	4.76 ± 0.20	5.14 ± 0.61	n.s.
Aspartic acid	161.9 ± 8.58	182.5 ± 20.8	n.s.
Glutamic acid	859.2 ± 39.0	807.1 ± 46.6	n.s.
Glutamine	486.4 ± 33.1	490.7 ± 63.9	n.s.
Glycine	65.2 ± 3.70	79.78 ± 9.64	n.s.
GABA	149.7 ± 8.07	177.3 ± 19.9	n.s.
Threonine	16.9 ± 1.50	19.0 ± 2.46	n.s.
Serine	64.9 ± 3.46	74.6 ± 8.90	n.s.
Alanine	69.9 ± 3.21	76.6 ± 8.53	n.s.
Cysteine	0.38 ± 0.04	0.39 ± 0.06	n.s.
Methionine	2.83 ± 0.26	4.01 ± 0.45	< 0.05
Arginine	6.76 ± 0.56	7.71 ± 1.17	n.s.
Lysine	4.42 ± 0.34	9.51 ± 0.48	n.s.
Total	1734.7 ± 90.3	1824.8 ± 112.7	n.s.
EAA	52.09 ± 3.06	76.57 ± 9.93	< 0.005
EAA-tryptophan	52.02 ± 3.50	56.99 ± 7.84	n.s.
LNAA	23.37 ± 1.51	42.66 ± 6.06	< 0.01
Tryptophan/LNAA(%)	0.2 ± 0.01	45.6 ± 3.9	< 0.0001

Brain amino acid composition in adult *Tdo*$^{-/-}$ mice was determined with an amino acid analyzer. Values represent means ± S. E. (nmol/100 mg tissue). EAA, valine, threonine, tryptophan, methionine, phenylalanine, leucine, isoleucine, and lysine; LNAA, tyrosine, phenylalanine, leucine, isoleucine, tryptophan, and valine; n.s., not significant.

Figure S1. Kanai *et al*.

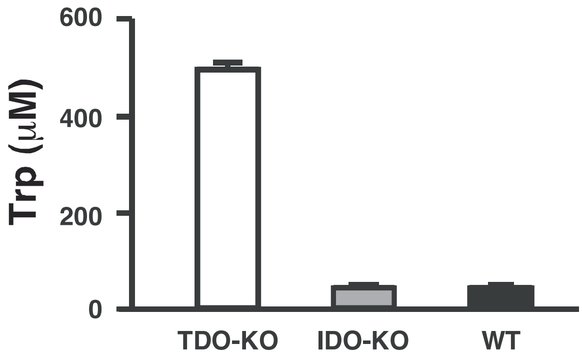

Figure S2. Kanai et al.

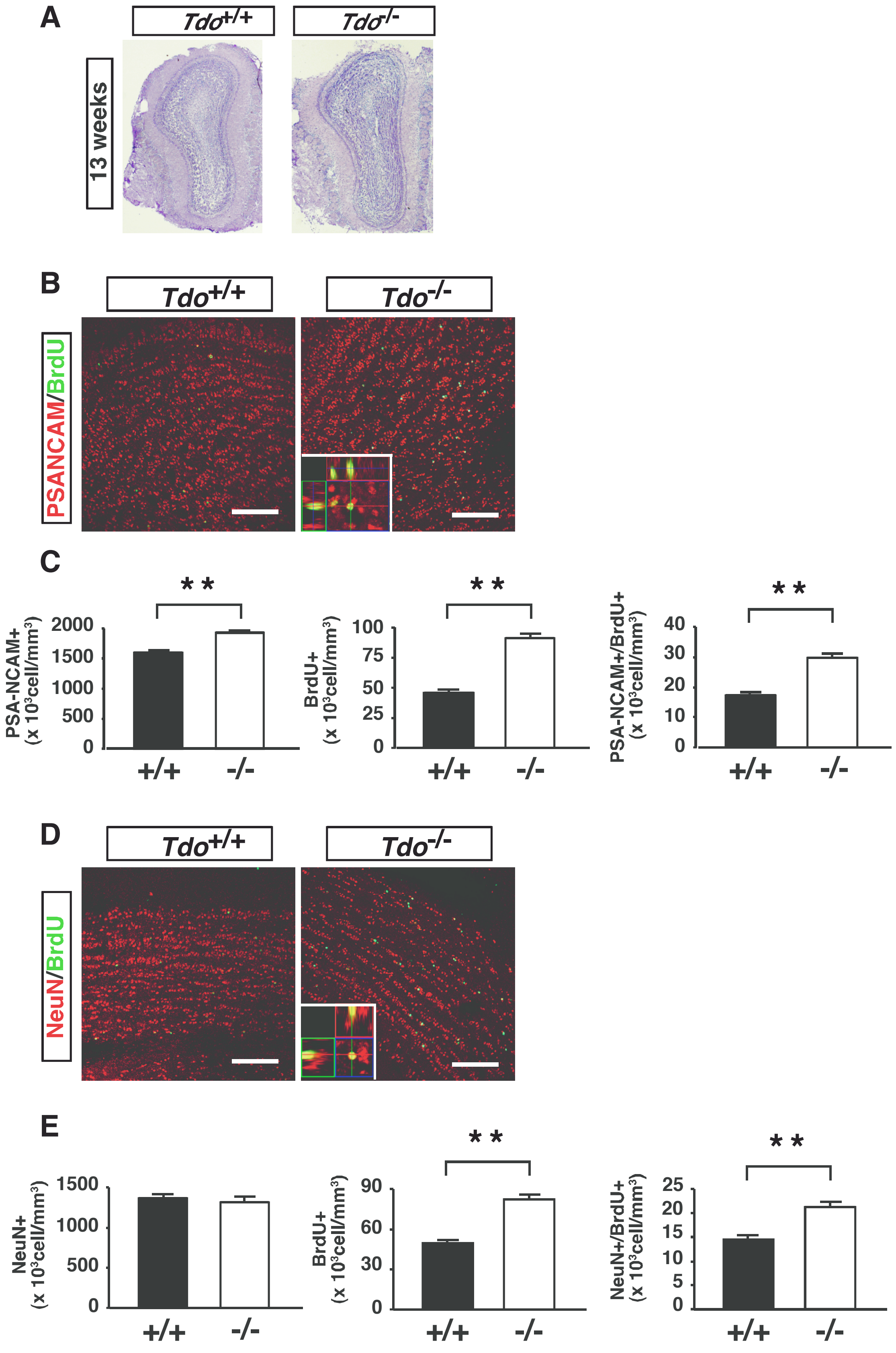

Figure S3. Kanai *et al*.

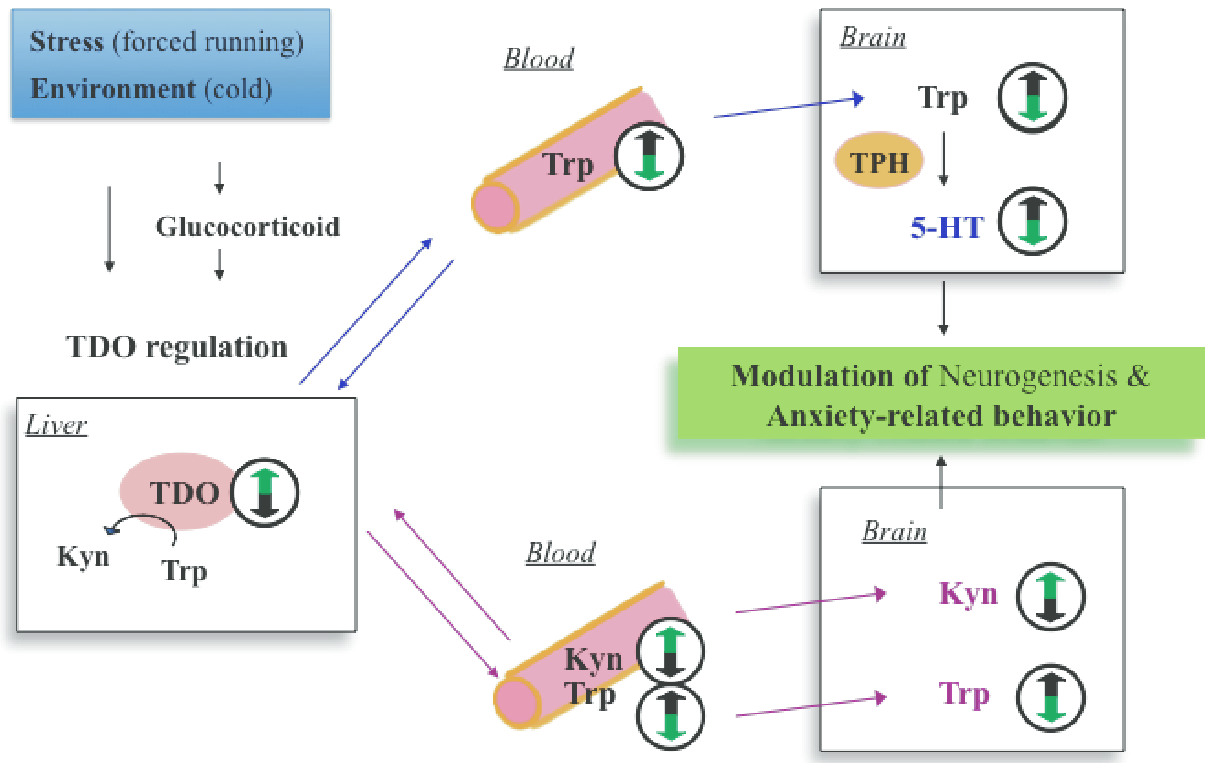

Supplement: Additional file 1 — Supplementary methods, Supplementary Tables S1-S2, and Supplementary Figures S1-S3 are included in this file. [file 1756-6606-2-8-S1.pdf]
